# Implementing information and communication technology education on food allergy and anaphylaxis in the school setting

**DOI:** 10.1002/clt2.12039

**Published:** 2021-07-03

**Authors:** Paloma Poza‐Guedes, Ruperto González‐Pérez

**Affiliations:** ^1^ Allergy Department Hospital Universitario de Canarias Tenerife Spain; ^2^ Severe Asthma Unit Hospital Universitario de Canarias Tenerife Spain

**Keywords:** anaphylaxis, educators, eHealth, eLearning, food allergy, management, school setting

## Abstract

**Introduction:**

Every year, 1/10,000 children experiences a food‐anaphylactic reaction. Most of these events, including attack‐related deaths, may happen during the school hours. In the current study, we assessed the influence of information and communication technologies (ICT) in the school‐staff's education on food allergy and anaphylaxis (FAA).

**Methods:**

The target population of this intervention was non‐university teaching centers from the local Regional Education Council, including both state and private institutions. The digital intervention was supported by the free‐of‐charge and open‐source learning‐management Aulatic Educational Platform. Structured questionnaires were developed to evaluate the educators' knowledge, feelings, and self‐efficacy on FAA, in addition to a satisfaction and quality survey of the training program.

**Results:**

A total of 1748 school‐educators were virtually enrolled from May 2016 to June 2020 in one of the 8‐week course editions, with 80.6% of attendees successfully completing the full training. All scores concerning school‐staff's basic knowledge and self‐efficacy on FAA significantly improved after the educational intervention, reaching a high level of satisfaction among participants (98.5%) over the 4‐year educational program.

**Conclusion:**

Our results highlighted the effectiveness of a focused e‐learning activity to improve teachers and school caretakers in the management of food allergic scholars and anaphylactic reactions during the school hours. The use of ICTs tools should become an integrated part of curricular frameworks in non‐university education, leading to a better care of FAA school children.

## 1 INTRODUCTION

As the access to and utilization of computers and mobile devices has increased, Internet usage has dramatically risen, placing an unprecedented amount of health information within reach of general consumers.^1^, ^2^ Electronic health (eHealth) embraces a diverse group of computer‐based technologies to improve the efficacy and efficiency of the health care industry. The application of eHealth not only refers to technological advances but also to a commitment of networked, global thinking to improve health care worldwide.^3^, ^4^, ^5^, ^6^ Information and communication technologies (ICTs) are a diverse range of Internet resources that include websites, health apps, podcasts, online interactive programs, and health‐related forums. These ICTs can contribute to broadening access to education, improving equity in education, the delivery of quality learning and teaching, teacher professional development, and more efficient education management, governance, and administration.^7^, ^8^

Food allergies (FAs) have been recognized as a public health burden in developed countries and are the leading cause of anaphylaxis in community health settings, affecting 2%–8% of people under 18 years of age throughout the world.^9^, ^10^, ^11^ FAs and anaphylaxis have a markedly negative impact on one's psychological well‐being, with a disproportionately lower quality of life found in allergic children and their families compared with their non‐allergic peers.^12^, ^13^, ^14^ The pathophysiology of FAs involves several immunological mechanisms that drive reactions, most commonly as immediate hypersensitivity to ingested antigens, in which specific IgE antibodies bound to mast cells and basophils lead to explicit physiological responses in target tissues.^15^ Nearly 18% of children have experienced an allergic reaction at school,^16^, ^17^ with 25% of first‐time anaphylactic reactions occurring during school hours; thus, it is necessary for educators to promptly recognize and deal with these unexpected events.^18^

Web‐based guidance provides a platform for health professionals to access flexible education to improve awareness, knowledge, and skills in delivering FA and anaphylaxis care.^19^ Although many educators have received information on FAs, previous studies have shown this information to have little influence on the outcomes of questionnaires. Additionally, it is possible that participants might attend first‐aid courses that fail to provide training on FAs and anaphylaxis, as adrenaline is often not administered, which increases the risk of hospitalization and death.^20^, ^21^

In Spain, school nurses have only recently^22^ been included in by‐laws as permanent staff, but they are not present in all country school facilities; therefore, the management of most students with food allergies depends almost entirely on school personnel with limited medical skills. To address this situation, in 2015, the Ministries of Health and Education, Culture & Sports of Spain published the “National Guidelines for Food and/or Latex Allergic Schoolchildren,” which allowed specific computer‐based food allergy education for schoolteachers.^23^

There is limited research on the effectiveness of educational interventions (EIs) for FAs, and the quality of eHealth resources is uncertain because the developers of eHealth instruments often have no health care training, and health professionals are generally not involved in the design of these tools. Thus, there is a demand for the school and the health system to improve their preparedness to handle students with FAs.^24^ In this regard, although eHealth interventions such as computer‐based applications, telecommunications, and mobile applications have delivered significant improvements for asthma patients in terms of improving inhaler technique, adherence, and quality of life, evidence for other chronic allergy conditions such as FA and FA anaphylaxis (FAA) is lacking.^25^ Previous studies have shown the effectiveness of brief, specific training courses for school staff and parents of children with FA,^26^, ^27^, ^28^ but the potential impact of extended eHealth learning on this topic has not been fully assessed. In the current study, we investigated the impact of an eHealth EI, which is supported by board‐certified allergists, on educators' knowledge and management of FAA over a 4‐year real‐world experience within different school settings in our community.

## 2 METHODS

### 2.1 Study population

The target population of this intervention was teachers who worked in non‐university educational centers in the Canary Islands Education Council, which covers 1134 teaching facilities (843 state and 291 private institutions) and includes an educational body of 28,377 teachers.^29^ An open virtual call. (https://www3.gobiernodecanarias.org/medusa/campus/aulatic/) was first made in April 2016 to teachers and principals from nursery school to high school levels in the Canary Islands, Spain. The project team included allergists from the Public Tertiary Care Community Hospital, computer operator assistants, and technicians from two local Councils of Health and Education, Universities, Culture & Sports from the Canary Islands.^30^ The study was reviewed and approved by the ethics committee of the Hospital Universitario Nuestra Señora de Candelaria on July 2, 2016 (reference numbers PI‐35/11 & 24/14). Informed consent was obtained from all participants, following the guidelines outlined in the Declaration of Helsinki.

### 2.2 Educational materials and interventions

**FIGURE 1:**
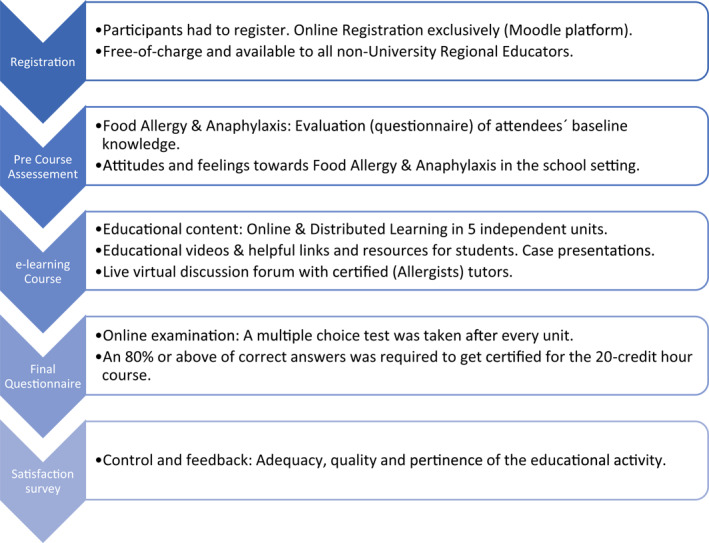
Digital journey map of the “Food Allergy & Anaphylaxis in the School Setting” educational intervention

The digital intervention was supported by the Aulatic Educational Platform, based on Moodle. Moodle is a free, open‐source, on‐demand learning management software and included the following (Figure 1):

Free *registration*: To take part in the online activity, participants must register online.
A *specific questionnaire* (supplementary material A) explored the teachers' baseline knowledge, attitudes, and feelings on FAA before taking the EI.
*Electronic learning* (*e‐learning) intervention*: Five educational units comprising downloadable board‐certified FA instructional material were specifically developed for the intended activity, covering the following topics:
  1. Introduction to allergies in the local school setting.
  2. Definitions and basic concepts of food and latex allergies, with descriptions of the involved allergic mechanisms.
  3. Signs and symptoms of FAs and anaphylaxis.
  4. Treatment and management of FA reactions.
  5. Development and explanation of a detailed FA emergency action plan in the participating school or institution.

Each educational unit remained available online for 2 months, allowing users to log in and keep up with the educational tools at any time. The contents could be revisited at will. A digital library contained 5‐min videos that discussed the following subjects: identification of FAA clinical symptoms, development of an individual FA plan for the classroom, and adrenaline self‐administration. A virtual *forum* section moderated by allergists allowed participants to comment and ask questions at any stage of the intervention. A final *written examination* and topic‐specific questionnaires were developed. A minimum score of 80% was needed to pass each educational unit and gain access to the subsequent e‐learning content. To obtain official certification for this 20‐h continuing education course, participants had to pass the final written examination.

A *post‐questionnaire* (supplementary material A) was used to investigate potential differences in the proportions of correct and incorrect answers to comparing the progress upon completion of the EI.
A mandatory EI *satisfaction survey* was administered to all participants to evaluate the following items:
  - Ability of the activity to accomplish the planned objectives.
  - Quality and quantity of the educational content.
  - Pertinence of e‐learning regarding the participant's current situation.
  - Translational changes to daily teaching practice (i.e., gaining competence to handle an unexpected FA situation within the educational setting, including administering medications).

An anonymous analog scoring system was developed to assess each of the aforementioned items (0 = Very poor, 5 = Excellent). In accordance with local regulations, attendants were initially advised that all the collected data would be kept anonymous and used to investigate the internal efficacy and quality of the e‐learning tool.

### 2.3 Statistics

A descriptive statistical analysis was conducted. For the data analysis, the pre‐program and post‐program questionnaire answers were compared. A *t*‐test and chi‐squared test were used to detect statistically significant differences in the proportions of correct and incorrect answers before and after the intervention. Statistical significance was set at *p* < 0.05.

## 3 RESULTS

### 3.1 Target population

A total of 1748 teachers attended at least one of the bi‐monthly online courses (five per school year) from May 2016 to June 2020 (20 digital editions), representing 6.16% of all regional non‐university educators in the Canary Islands.^29^ All participants anonymously filled out the pre‐course questionnaire. A total of 1409 participants (80.6%) completed the full program, with a dropout rate of less than 20% over the 4‐year study period.

Participants from across professional degrees were included in the study (Table 1) included the following:

‐ Teachers (80.28%): elementary (40.86%) and secondary education (29.08%)
‐ Teaching supporting teams (12.9%) including licensed special educators (i.e., those who are trained to meet the needs of students with disabilities) and educational psychology professionals.
‐ State officials (6.8%): management and executive teams

**TABLE 1:** A total of 1748 school‐educators attended online one of the “Food Allergy & Anaphylaxis in the School Setting” editions from May 2016 to April 2020

**Teachers (*n* = 1403)**	**80.28%**
Nursery	16.18%
Elementary school	24.67%
Secondary school	29.08%
Vocational education	17.94%
Language schools	1.2%
Adult teaching	1.2%
Arts	1.28%
Others	8.41%

Supporting teams (*n* = 225)	12.9%
School counselors & psycopedagogist	0.8%
Special educational needs	3.12%
Others	8.9%

State officials (*n* = 117)	6.73%
Executive officers and education managers	1.52%
Local coordinators	3.68%
Others	1.21%

*Note:* Participant´s hierarchy and professional degrees are shown.

Regarding the type of teaching institutions, the participants were distributed as follows:

‐ Ordinary education: nursery (16.18%), elementary (24.67%), and secondary education (including high school) (29.08%)
‐ Vocational education (17.94%)
‐ Supplementary education teachers from official language schools, art schools, and adult teaching programs (12.09%)

Although the majority of the represented educational institutions were public, 7.6% of the participants worked in subsidized charter schools.

### 3.2 Trends in registration

**FIGURE 2:**
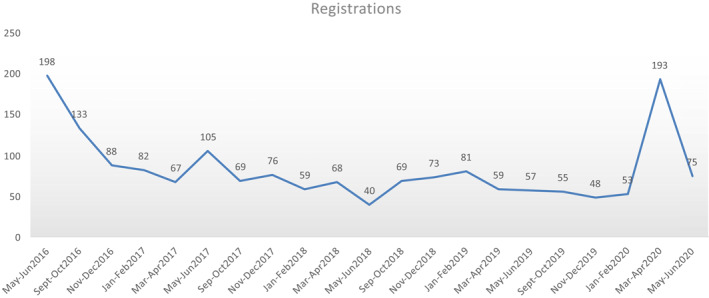
Trends in registration numbers for the virtual educational intervention entitled “Food Allergy & Anaphylaxis in the School Setting” from May 2016 to June 2020. A marked increase in the registration number was noticeable during the March–April 2020 edition, during the mandatory COVID‐19 quarantine in Spain

The total number of bi‐monthly registered participants was 198 in the first EI edition in May 2016, followed by a median of 79 attendees per course for the subsequent 4 years. There was a marked increase in the registration number (up to 193 participants) during the March–April 2020 edition, which occurred during the mandatory COVID‐19 quarantine in Spain (Figure 2).

**FIGURE 3:**
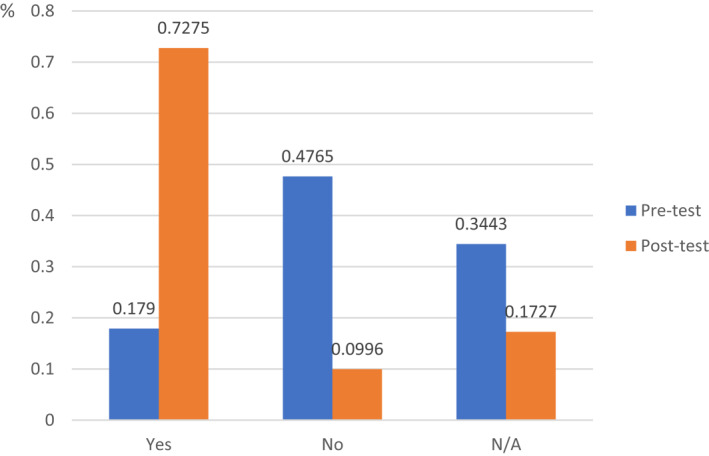
Evaluation of structured questionnaires, concerning self‐competency in the correct management of a food allergy reaction and anaphylaxis in the school setting, before and after the educational intervention

### 3.3 Questionnaire assessment

The pre‐ and post‐EI surveys assessed the individual knowledge and awareness of FA and anaphylaxis in the school setting and covered the following topics:

‐ Motivation to accomplish the EI: In the pre‐EI survey, more than half of the participants confirmed that the main reason for registering for the EI was “personal interest in FAs and facing the needs of FAs in the school setting.”
‐ Awareness of FAs in the school setting: 81.82% of the participants thought that FAs were “troublesome for the school staff”, while 64.37% agreed that FAs were mainly an issue of concern for the students. Upon completion of the EI, up to 90% (*p* < 0.01) of participants stated that FAs were a “worrying complaint in the educational background.” Before taking part in the EI, 86% of the participants considered FAs as an “issue to be exclusively managed by the student's family and/or close relatives”; this changed to 60% after completion of the EI (*p* = 0.01). In addition, 12.3% of all participants reported previous involvement in a FA emergency situation, with 3.6% of participants being involved in a documented anaphylactic event during school hours. More than one‐third of participants stated that proper management by the institution (36.7%) and their personal contribution (34.9%) aided in the final resolution of the allergic episode.
‐ Evaluation of self‐competence regarding the correct management of a FA reaction in the school: In the pre‐EI survey, only 17.9% of the participants considered themselves capable of actively delivering aid of any kind in the event of a severe FA reaction during school hours. Although only 28.45% of the participants knew about the use of adrenaline auto‐injectors, up to 60% (*p* = 0.01) stated that they would certainly administer this medication to a student under specific circumstances (i.e., in a severe or rapid FA reaction). According to the post‐EI survey, 72.78% of the participants felt “confident in facing an unexpected FA situation within the educational facility” (Figure 3), increasing from an initial 17.9% upon accomplishment of the EI (*p* < 0.001).

### 3.4 Quality and overall satisfaction assessment of the EI

A final post‐course survey was conducted to evaluate the quality and appropriateness of the activity through a Likert score (0 = Very poor; 10: Excellent). An overall evaluation above eight points was recorded by 90.62% of the participants, and 99.62% stated that they would definitely recommend the EI to a colleague. In addition, 93.4% of participants stated that they would implement changes in their teaching daily practice upon completion of this specific training, with a score of greater than four points (0 = Very poor, 5 = Excellent) from virtually all (98.5%) participants.

## 4 DISCUSSION

Similar to reports from other countries, Spain has seen marked increases in the rates of FA and anaphylaxis over the last decade.^31^, ^32^ Only approximately two‐thirds of patients with prior anaphylaxis had a prescribed epinephrine autoinjector device available at the time of their subsequent anaphylactic event, contributing to a 35% hospital admission rate for the same medical emergency.^33^ As allergic reactions may occur anywhere during school hours, including in the classroom, lunchroom, playground area, on fileld trips, and traveling to and from school, educators are frequently the first adult to respond to these unpredictable events.^34^

Although the development of self‐efficacy, defined as the belief that an individual can carry out a behavior necessary to reach an expected outcome, has been described as a key factor in improving medical care,^35^ it remains unclear whether self‐efficacy is related to the management of FA among schoolteachers.^36^ Today, the adoption of ICT by educational staff is an ongoing process, developing from acknowledging the possibilities of ICT in education to further evolved creative uses of technology for teaching and learning.^7^ However, we believe that the use of ICT has not been sufficiently utilized as a valuable tool to improve educators' skills and to provide effective guidance regarding FA and anaphylaxis in a school context. Although there have been diverse educational strategies to prevent, recognize, and apply proper treatment of allergic reactions,^37^ studies have shown that school staff are still insufficiently prepared to deal with FA events, including the administration of adrenaline.^38^, ^39^ However, Shah et al. found that even a 1‐h educational intervention significantly increased teachers' knowledge of FA causes, symptoms, and treatment of reactions in educational settings with different socioeconomic situations.^40^ Additionally, Ruíz‐Baqués et al.^41^ found that parents' and caregivers' understanding of children with FAs improved by 50% after taking an online FA educational program. González‐Mancebo et al. confirmed the usefulness of a self‐efficacy scale for teaching and cafeteria staff to assess the management of food allergies and anaphylaxis, especially concerning the early administration of adrenaline.^42^

The present 8‐week digital program significantly improved participants' self‐efficacy in the management of FAs and anaphylaxis, which increased from 17.9% to 72.78%. The self‐efficacy was higher than after previous interventions that used FA‐focused peer educational videos (23.8%)^43^ or shorter (2‐week) FA‐training schedules (50%).^41^ This online EI program is a viable and exciting method for instructional delivery, providing students with a flexible schedule that allows them to access the material at any time and work at their own pace, avoiding the need to commute to an educational facility. Although not quantified in the present work, the ability to remotely access the courses is a prominent benefit of this digital EI, which is intended to reach dispersed territories, including eight different islands. Remote access saves transportation expenses such as airline and boat fares that are often necessary for accessing in‐person training. The high enrollment rate of nearly 1700 participants and a substantial completion rate (approximately 80%) was found for the current EI across the 4‐year study period, highlighting the interest and demand for quality FA e‐education as a practical training in the school setting. Interestingly, the demand for this digital EI doubled during the mandatory COVID‐19 confinement in Spain, indicating the participants' willingness to devote time to self‐education and acquire new professional skills.

An effective and functional web‐based teaching program has previously been defined as one that measures results while being accessible, user‐friendly, and able to transmit information that can be translated into professional practice.^44^, ^45^ In the present investigation, the web‐based learning platform Aulatic effectively provided the specific FA educational training, enhancing the learner's knowledge, skills, and attitudes regarding the delivery of school‐centered care. An encouraging achievement of the investigated EI is the significant overall post‐course improvement (above 54%) in the participants' self‐perception to implement changes in their daily practice regarding students with FAs. In line with previous reports, the areas of knowledge where participants showed the lowest self‐competence were the recognition of symptoms and the treatment of allergic reactions, especially regarding the administration of adrenaline.^46^, ^47^, ^48^ Our results showed a sustained and significant increase throughout the 4‐year duration of the program in the scores concerning FA knowledge, including the number of participants stating that anaphylaxis could be properly managed at their school.

Some limitations of this study should be mentioned. Because the included participants voluntarily attended the course, it is possible that they were more likely to develop increased self‐efficacy since they may have been more willing to learn the material.^38^ Further studies that include a control group may clarify whether compulsory EIs would have similar outcomes. It would also be interesting to investigate the durability of the increased self‐efficacy after participants complete the digital EI. Finally, voluntary enrolment and low awareness and/or limited knowledge of the new national legislation regarding food allergy education at school may explain the final number of enrolled participants.

The current EI follows the work by Voogt et al.^7^ which showed that effective professional ICTs need to focus on translating general ideas into specific classroom applications through scholar‐centered EIs. These EIs should expose participants to actual practice by providing real‐case presentations rather than descriptions; provide opportunities for group support through the associated open virtual forum; and finally, evaluate participants and provide feedback from skilled practitioners, such as a professional allergist.

## 5 CONCLUSION

The present EI intended to meet the information needs of teachers and other school professionals by addressing the complex issue of FAs and anaphylaxis in the school setting, which is exacerbated by fear, lack of knowledge, and poor training. However, many educators are willing to learn more about these conditions. The accessibility to the virtual EI, the high quality of the content (which was an officially certified training program), and the provision of comprehensive coaching were crucial to enabling a large number of participants to successfully complete the EI.

Although constant improvement to overcome limitations is warranted, such as obtaining evidence on the effectiveness in terms of pedagogical parameters,^49^, ^50^ eHealth applications have become a compelling educational tool, necessitating the continued exploration of these contemporary technologies. Digital mentoring from allergists through ICT learning tools may be both effective and stimulating, enhancing educators' understanding and self‐efficacy to ensure safer and socially inclusive FA management in schools.

## CONFLICT OF INTEREST

All authors declare that there is no conflict of interest regarding the publication of this manuscript.

## AUTHORS' CONTRIBUTIONS

**PPG** and **RGP** designed the study and wrote the manuscript. PPG completed the data collection and RGP performed the literature search. All authors contributed equally to the critical interpretation of the results. All authors approved the final version of the manuscript.

## Supporting information

Supplementary Material
