# Supplementary material for: Implementing information and communication technology education on food allergy and anaphylaxis in the school setting
Source: Clin Transl Allergy. 2021 Jul 3;11(5):e12039. doi: 10.1002/clt2.12039 (PMC8254581; doi:10.1002/clt2.12039)
Supplement: Supplementary file 1 — Supplementary Material [file CLT2-11-e12039-s001.docx]

***Food Allergy and Anaphylaxis on-line Questionnaire: Teachers***

**Please select the most appropriate response. All answers will remain confidential.**

**1. What is your motivation to take part in this educational activity?***

a. Lack of knowledge dealing with food allergies, as I have not been previously trained in this medical condition

b. I have experienced previous food allergic reactions during the school hours

c. I deal with food allergic students in my daily professional activity

d. Personal interest in food allergy to face the needs of this condition in the school setting

e. None of the above (please state reason):

**2. As a teacher, do you believe food allergy is a worrying complaint in the educational background?**

a. Yes

b. No

c. Don´t know/not sure

**3. As a teacher, do you believe food allergy is nowadays a troublesome medical condition limited to the schoolchildren/students?**

a. Yes

b. No

c. Don´t know/not sure

**4. As a teacher, do you believe food allergy is a troublesome medical condition limited to those families/relatives with allergic for schoolchildren/students?**

a. Yes

b. No

c. Don´t know/not sure

**5. How familiar are you with food allergies?***

a. Very unfamiliar

b. Unfamilliar

c. Somewhat familiar

d. Familiar

e. Very familiar

**6. Have you ever been involved in the management of a severe food allergic reaction (anaphylaxis) in your workplace?***

a. Yes

b. No

**7. If so, was your personal involvement of any help to overcome or improve the situation during the food allergy reaction? ***

a. Yes

b. No

c. N/A

**8, Did you experience any concern while dealing with such food allergy reaction/s?***

a. Yes

b. No

c. N/A

**9. Do you feel properly trained and ready to face an unexpected food allergy reaction in your workplace at present?**

a. Yes

b. No

**10. Do you feel confident to give an adrenaline injection to one of your schoolchildren/student eventually afflicted with a severe food allergic reaction at present?**

a. Yes

b. No

c. N/A

**Thank you very much for completing this survey.**

****Pre-interventional questionnaire only.***
